# Physical exercise-induced mental health benefits in future physicians: a dual-chain mediation of peer support and professional identity formation

**DOI:** 10.3389/fpsyg.2025.1640506

**Published:** 2025-12-03

**Authors:** Kaiqi Liu, Kaili Pan, Dan Liang

**Affiliations:** 1 Department of Physical Education, Fujian Medical University, Fuzhou, China; 2 Department of Physical Education, China University of Geosciences, Beijing, China

**Keywords:** peer support, mental health, physical exercise, medical students, mediation analysis

## Abstract

**Objective:**

This study investigates the dual chain mediating roles of peer support and professional identity formation in the relationship between physical exercise and mental health among medical students.

**Methods:**

A cross-sectional survey was conducted on 420 medical students in China, using structural equation modeling (SEM) to test hypothesized pathways.

**Results:**

Exercise frequency demonstrated a significant direct association with better mental health (β = 0.28, *P* < 0.001). Two distinct mediating pathways were supported: (1) a significant serial mediation chain (exercise → peer support → professional identity → mental health; β = 0.044, *P* < 0.001) and (2) a significant parallel mediation chain (exercise → professional identity → mental health; β = 0.074, *P* < 0.001). Multi-group analysis revealed that the mediating role of professional identity was significantly stronger in clinical-year students (e.g., path from exercise to professional identity: Δβ = 0.10, *P* = 0.005).

**Limitations:**

The cross-sectional design precludes causal inference, and the cultural and professional specificity of the sample may affect generalizability.

**Conclusion:**

These findings highlight the complex psychosocial mechanisms through which exercise is associated with enhanced mental health in future physicians, with implications for targeted intervention design.

## 1 Introduction

Medical students face unique psychological challenges, including high rates of burnout (55–75%), anxiety (25–30%), and depression (15–20%) (Halperin et al., 2021; Harolds, 2021; Liu et al., 2023). These mental health issues are closely linked to academic pressure, clinical exposure to suffering, and the professional identity formation process (Jones et al., 2023; Mirza et al., 2021; Pokhrel et al., 2020). Physical exercise has emerged as a promising non-pharmacological intervention, with meta-analyses showing it reduces anxiety and depression symptoms with medium-to-large effect sizes (Tang et al., 2022; Zheng et al., 2024). However, most studies focus on direct physiological effects (e.g., endorphin release), neglecting the social and identity-related mechanisms through which exercise may operate in professional training contexts (Davis et al., 2021; Schoenfeld and Swanson, 2021). Group-based physical activities (e.g., team sports, fitness classes) offer dual benefits: physical exertion and social interaction (Inoue et al., 2024; Novak et al., 2025). For medical students, these activities may foster peer support networks and reinforce professional identity—two critical protective factors in high-stakes educational environments (Beck-Pancer and Kryzhanovskaya, 2025; Bremer et al., 2022). Peer support buffers stress by providing emotional validation and practical assistance, while professional identity (defined as a sense of belonging to the medical profession and confidence in clinical roles) mitigates burnout by aligning personal values with career goals (Luo et al., 2024). Despite the established benefits of physical exercise for mental health and the recognized importance of peer support and professional identity in medical education, a significant gap remains in understanding the psychosocial mechanisms that link these constructions. Previous research has largely focused on either the direct physiological effects of exercise or the independent roles of social support and identity formation. No study has empirically tested an integrated model that examines how peer support and professional identity sequentially and jointly mediate the relationship between exercise and mental health in medical students. This study aims to fill this gap by proposing and testing a dual-chain mediation model, which posits that exercise not only directly improves mental health but also operates through two distinct pathways: a serial mediation chain (exercise → peer support → professional identity → mental health) and a parallel mediation chain (exercise → professional identity → mental health). By integrating principles from Stress-Buffering Theory, Social Identity Theory, and Self-Determination Theory, this research provides a comprehensive theoretical framework to elucidate the complex interplay between physical activity, social factors, and identity development in shaping the psychological wellbeing of future physicians.

### 1.1 Theoretical framework

#### 1.1.1 Stress-buffering hypothesis

Cohen and Wills’ theory posits that peer support alleviates stress by enhancing emotional resilience (Cohen and Wills, 1985). In exercise settings, shared goals (e.g., training for a race) and collaborative dynamics create micro-communities where medical students can discuss professional challenges, reducing feelings of isolation. For example, a group cycling program may provide opportunities to debrief after clinical rotations, strengthening peer bonds. Research has revealed a significant positive correlation between peer support and psychological resilience (*r* = 0.226, *P* = 0.00) (Karadas and Duran, 2022). Moreover, resilience plays a mediating role in the relationship between peer support and job stress. For instance, in the male population, the alleviation of stress by peer support is entirely mediated by resilience. In contrast, in the female population, the mediating effect is partial but stronger. This suggests that peer support reduces perceived stress by enhancing individuals’ inherent capacity to cope with stress, namely resilience (Yalcin-Siedentopf et al., 2021). Emotional resilience not only mediates the relationship between peer support and stress but also moderates the negative impact of stress on mental health. For example, during the COVID-19 pandemic, emotional resilience significantly moderates the relationship between perceived stress and social adaptation. Meanwhile, peer support reduces anxiety and depression symptoms through resilience, with the mediating effects accounting for 30.9 and 20.9%, respectively (Hu et al., 2023; Yan et al., 2025). This theoretical framework provides the foundation for our hypothesis that peer support plays a critical mediating role, specifically forming the initial link (Exercise → Peer Support) in our proposed serial mediation chain (H3).

#### 1.1.2 Social identity theory

Tajfel and Turner’s theory suggests that group memberships shape self-concept. When exercise groups are framed as part of professional training (e.g., “physician wellness teams”), participants may associate physical activity with their future roles as healers (Islam, 2014). Within such exercise-based micro-communities, peer support serves as a key mechanism that validates and reinforces this emerging professional identity. Through shared physical challenges and mutual encouragement, students not only bond as peers but also collectively enact and affirm the values and competencies associated with their future profession, such as teamwork, resilience, and mutual aid. This process accelerates professional identity formation, as seen in studies where team sports participation correlated with higher career commitment. In accordance with the bidirectional nature of peer support, both receiving and providing support within these activity contexts (e.g., encouraging a struggling teammate) can significantly enhance medical students’ sense of belonging and efficacy in their professional role. This kind of support indirectly promotes professional identity development through the chained mediating role of achievement, motivation and sense of meaning in life (Zaneb and Armitage-Chan, 2023). For example, nursing students can strengthen their professional identity through role models and socialization processes in the clinical environment (Willetts and Garvey, 2020). Thus, peer support within exercise groups is posited to be a catalyst for translating group membership into a strengthened professional self-concept. This theorizing directly underpins two of our hypotheses: that peer support mediates the relationship between exercise and professional identity (H2), and that it serves as a crucial preceding factor in the serial mediation pathway from exercise to mental health (H3).

#### 1.1.3 Self-determination theory

Ryan and Deci emphasize the role of social relatedness and competence in psychological wellbeing (Ryan and Deci, 2020). Peer interaction in sports can significantly enhance the sense of social connectedness, manifested as satisfaction in interpersonal acceptance and intimacy. Experiments have demonstrated that cooperative sports activities (such as team sports) are more effective than individual or competitive activities in enhancing intrinsic motivation, positive affect, and self-efficacy, while also promoting task engagement (Kaefer and Chiviacowsky, 2022). Group sports (such as team training or community activities) can reduce feelings of social isolation and improve mental health by providing emotional support, information exchange, and companionship. For example, group-based activities in college physical education courses can significantly enhance students’ social capital (such as trust and cooperation) (Gu et al., 2024). Peer coaching or collaborative training models (such as “completing sports challenges with peers”) have been proven to enhance the need for relatedness, thereby improving illness perception and quality of life (DeShazo et al., 2023). In conclusion, exercise satisfies the need for relatedness through peer interactions and builds competence via skill development, both of which are foundational to professional identity and mental health. The shaping effect on professional identity is strong when sports meet both needs simultaneously (e.g., team sports that provide peer support and promote skill development). For instance, nursing students develop professional identity through peer cooperation and skill development (Blackford et al., 2024), while art students enhance their mental health by improving relatedness and competence in collaborative creation (Ye et al., 2025).

#### 1.1.4 The unique role of peer support in identity formation

While Self-Determination Theory outlines the basic needs for relatedness and competence, the specific context of peer support within physical exercise offers a uniquely potent environment for professional identity formation in medical trainees. Unlike generic social interaction, peer support in shared physical activities often involves observational learning, vicarious experience, and normative social comparison related to professional behaviors and attitudes. For instance, witnessing a peer persevere through a difficult workout can metaphorically reinforce the value of perseverance in clinical practice. Discussions that naturally occur during or after group exercise can serve as informal “communities of practice” where students negotiate the meanings, values, and challenges of their future roles. Therefore, the peer support cultivated in exercise settings is not merely about fulfilling a need for relatedness; it actively provides identity-relevant feedback, role modeling, and collaborative sense-making, thereby directly feeding into the construction of a stable and positive professional identity. This delineates our model from those that position social support merely as a general wellbeing buffer, specifying its active, identity-shaping function within the high-stakes context of medical training. The mechanisms of relatedness and competence outlined here are central to our entire model, providing the theoretical rationale for the parallel mediation pathway where exercise directly enhances professional identity (H4), and for the final link where professional identity, in turn, promotes mental health (as part of both H3 and H4).

### 1.2 Proposed theoretical model

**FIGURE 1:**
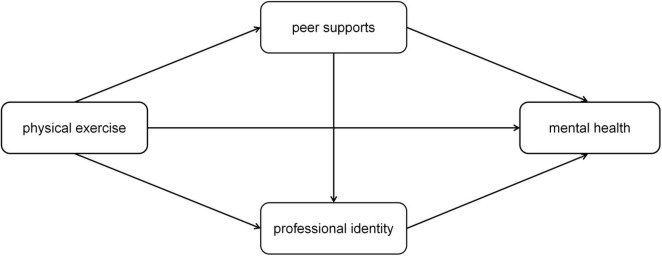
The hypothesized dual-chain mediation model linking physical exercise, peer support, professional identity, and mental health. H1: Direct effect of exercise on mental health. H2: Mediating role of peer support between exercise and professional identity. H3: Serial mediation (exercise → peer support → professional identity → mental health). H4: Parallel mediation (exercise → professional identity → mental health).

Based on the aforementioned theories, we propose a dual-chain mediation model to explain the relationship between physical exercise and mental health among medical students. As illustrated in Figure 1, the model encompasses both direct and indirect pathways. The indirect pathways include: (1) a serial mediation chain where exercise fosters peer support, which in turn enhances professional identity, ultimately leading to better mental health; and (2) a parallel mediation chain where exercise directly strengthens professional identity, thereby improving mental health. This model visually summarizes our four main hypotheses (H1–H4).

### 1.3 Research hypotheses

Guided by these theories, we tested four hypotheses (which are also summarized in Figure 1):

*H1:* Physical exercise frequency is positively associated with mental health.

While the direct physiological benefits of exercise are well-established, this hypothesis is grounded in Self-Determination Theory. We posit that in the high-stress context of medical training, the intrinsic sense of competence and autonomy gained from regular physical activity provides a direct counterbalance to academic and clinical stressors, thereby predicting lower burnout and higher psychological wellbeing, independent of social mechanisms.

*H2:* Peer support mediates the relationship between physical exercise and professional identity.

Drawing on Social Identity Theory, we argue that group-based physical activities (e.g., sports teams, fitness classes) create salient in-groups for medical students. Within these groups, shared experiences and goals foster a sense of belonging. We infer that this exercise-facilitated peer support serves as a platform for “socially validating” one’s emerging professional role, thereby strengthening professional identity. This extends the theory by applying it to informal, non-academic groups within professional training.

*H3:* Peer support and professional identity serially mediate the relationship between physical exercise and mental health.

This hypothesis integrates the Stress-Buffering Hypothesis and Social Identity Theory into a causal chain. We reason that peer support from exercise (Hypothesis 2) not only buffers stress but also cultivates a shared professional identity. A stronger professional identity, in turn, is theorized to enhance mental health by providing a coherent sense of purpose and meaning (Self-Determination Theory), which is crucial for resilience during challenging clinical training. This serial pathway tests a novel mechanism where exercise’s social benefits are channeled through identity development to impact wellbeing.

*H4:* Professional identity mediates the relationship between physical exercise and mental health.

We propose that exercise may also build professional identity through non-social pathways. Based on Self-Determination Theory, the process of setting and achieving personal fitness goals can enhance feelings of competence and self-efficacy. In the medical context, we argue that these feelings can metaphorically generalize and reinforce the competencies required in a clinical role (e.g., discipline, perseverance), thereby directly strengthening professional identity and subsequent mental health, even in individual exercise settings.

#### 1.3.1 Exploratory aim

The mediation pathways involving professional identity are stronger among clinical-year students than among pre-clinical students.

As students transition to the clinical phase, they face intensified patient-care responsibilities and role identity conflicts. We hypothesize that this context makes professional identity a more salient and powerful resource. Therefore, we expect the paths to professional identity (from exercise and peer support) and from professional identity to mental health to be significantly stronger for clinical-year students. This tests the context-dependent nature of our proposed model, a crucial consideration for designing stage-specific interventions.

## 2 Materials and methods

### 2.1 Study design

A cross-sectional design was used. Participants were recruited through a convenience sampling method. Specifically, study invitations containing a link to the online survey were distributed via official university email lists and student group chats across all five academic years of the medical program. Participation was voluntary, and informed consent was obtained electronically prior to survey completion. The inclusion criteria were: aged ≥ 18 years and currently enrolled in the medical school. A total of 420 medical students (238 females, 182 males; mean age = 22.7 ± 2.3 years) from pre-clinical (years 1–2, *n* = 252) and clinical (years 3–5, *n* = 168) programs. Inclusion criteria: aged ≥ 18 years, currently enrolled in medical school, and willing to provide informed consent. Exclusion criteria: history of severe mental illness or incomplete survey completion (response rate = 92%). The experimental protocol of this study has been approved by the Institutional Review Board of the Department of Physical Education, Fujian Medical University. All experimental procedures adhered to the ethical guidelines established by the committee, ensuring the ethicality of the research (World Medical Association, 2013). Informed consent was obtained from all participants prior to their participation. A total of 420 questionnaires were distributed and yielded 369 valid responses, which corresponds to an effective response rate of 97.8%. Detailed data are provided in Table 1.

**TABLE 1:** Demographic and study variable descriptives for a sample of Chinese medical students (N = 420).

Demographic characteristics	Male	Female	Total/proportion
Pre-clinical	121	131	252/60%
Clinical	61	107	168/40%
Total/proportion	182/43.3%	238/56.7%	420

### 2.2 Statistical analysis

#### 2.2.1 Preliminary analyses

Descriptive statistics (means, SD, frequencies) and Pearson correlations were calculated using SPSS 28.0. Composite scores for burnout (reverse-scored) and psychological wellbeing were created by averaging subscale scores.

#### 2.2.2 Structural equation modeling

Using Mplus 8.3, we tested a dual-chain mediation model with the following steps (Thoemmes et al., 2010): (1) Confirmatory Factor Analysis (CFA) to validate the four-factor structure (physical exercise, peer support, professional identity, mental health); (2) Path Model Testing to evaluate direct and indirect effects, with demographic variables (age, gender, academic year, clinical hours) included as covariates; (3) Bootstrap Resampling to estimate 95% bias-corrected confidence intervals (CIs) for indirect effects; (4) Multigroup SEM to compare mediation paths between pre-clinical and clinical students.

### 2.3 Measures

All instruments were administered in Chinese, with back-translation to English verified by bilingual experts to ensure cross-cultural equivalence. Constructs were measured using established scales with demonstrated reliability and validity in prior behavioral science research. The psychometric properties (Cronbach’s α, Composite Reliability, and Average Variance Extracted) for the multi-item scales in this sample are reported in Supplementary Table 1 and confirmed good reliability and convergent validity (see Supplementary Table 1).

#### 2.3.1 Physical exercise

Exercise behavior was assessed via the International Physical Activity Questionnaire-Short Form (IPAQ-SF), a validated tool for quantifying physical activity in adult populations (Hagströmer et al., 2006; Lee et al., 2011). Participants reported the frequency of moderate-to-vigorous physical activity (days/week), defined as activities lasting ≥ 10 min that increased heart rate or breathing (e.g., running, swimming, team sports). This single-item metric was chosen for its strong correlation with total physical activity scores in student samples (*r* = 0.82, *P* < 0.001) and practical relevance for intervention design.

#### 2.3.2 Peer support

Perceived peer support was measured using the 8-item Multidimensional Peer Support Scale (MPSS), adapted from the original 12-item version to focus on exercise-related social connections (Chou, 2000; Sun and Guo, 2024). Sample items included: “I can rely on my peers for encouragement during physical activity” and “My peers and I share strategies to balance exercise and study schedules.” Responses were rated on a 5-point Likert scale (1 = strongly disagree, 5 = strongly agree). The scale demonstrated acceptable internal consistency in this sample (Cronbach’s α = 0.86), consistent with prior studies in educational settings (α = 0.81–0.89).

#### 2.3.3 Professional identity

Professional identity formation was evaluated using the 10-item Medical Professional Identity Scale (MPIS) (Sarraf-Yazdi et al., 2021; Tagawa, 2019). For medical trainee populations. Items assessed self-identification with medical roles (e.g., “I feel proud to be training as a physician”) and confidence in clinical competencies (e.g., “I am capable of handling challenging patient interactions”). Responses used a 7-point Likert scale (1 = strongly disagree, 7 = strongly agree), with higher scores indicating stronger professional identity. The scale showed excellent reliability (α = 0.91) and convergent validity (*r* = 0.63 with career commitment measures, *P* < 0.001).

#### 2.3.4 Mental health outcomes

Demographic information such as age, gender, academic year, and weekly study hours was also collected. Age was recorded as a continuous variable, while gender was categorized as male or female. The academic year was divided into pre-clinical years (first- second year) and clinical-year (third-fifth year). Weekly study hours were self-reported and treated as a continuous variable.

1. Burnout: The Maslach Burnout Inventory-Student Version (MBI-SV) was used to measure emotional exhaustion, depersonalization, and personal accomplishment (Pérez-Mármol and Brown, 2019; Yavuz Temel and Doğan, 2014). A composite burnout score was created by reverse-scoring exhaustion and depersonalization items and averaging across subscales (higher scores = better mental health). Cronbach’s α for the composite score was 0.89.
2. Psychological Wellbeing: The Ryff’s Scales of Psychological Wellbeing assessed six dimensions (self-acceptance, positive relations, autonomy, environmental mastery, purpose in life, personal growth) (Babnik et al., 2021; van Dierendonck and Lam, 2023). Items were averaged to form a global wellbeing score (range: 1–6), with higher scores indicating greater wellbeing (α = 0.92).

#### 2.3.5 Demographic and contextual variables

Age (continuous variable, years), gender (male/female), academic year (pre-clinical [years 1–2]/clinical [years 3–5]), and weekly clinical hours (self-reported, continuous variable) were collected as covariates. Clinical hours were included to account for differential stressors between training stages, as prior research links direct patient contact to increased burnout.

#### 2.3.6 Data quality control

To minimize common method variance, the survey included (Kock et al., 2021):

1. A reverse-worded validity item (“I sometimes skip answering survey questions randomly”) to identify careless responders (*n* = 8, excluded).
2. Item randomization across sections to reduce response set bias.
3. A single-informant bias statement in the consent form, emphasizing the study’s exploratory nature and encouraging honest responses.

## 3 Results

### 3.1 Sample characteristics

Table 2 presents demographic and variable descriptives. Most participants were pre-clinical students (60%), with an average exercise frequency of 3.1 days/week. Burnout levels were moderate (*M* = 3.2 ± 0.8), while psychological wellbeing was above average (*M* = 4.5 ± 0.9). Peer support and professional identity were positively correlated with exercise frequency (*r* = 0.41, *P* < 0.001; *r* = 0.35, *P* < 0.001) and negatively correlated with burnout (*r* = -0.45, *P* < 0.001; *r* = -0.52, *P* < 0.001).

**TABLE 2:** Descriptive statistics and Pearson correlations for key study variables.

Variable	Mean (SD)	Range	Correlation with exercise frequency
Age (years)	22.7 (2.3)	18–28	0.12*
Weekly clinical hours	12.5 (8.4)	0–30	−0.09
Exercise frequency (days/week)	3.1 (1.6)	0–7	–
Peer support	3.8 (0.9)	1–5	0.41***
Professional identity	5.1 (1.2)	1–7	0.35***
Burnout (reverse-scored)	4.8 (1.1)	1–7	−0.37***
Psychological wellbeing	4.5 (0.9)	1–6	0.39***

n = 420. Burnout score was reverse-scored so that higher values indicate better mental health.

*P < 0.05, **P < 0.01, ***P < 0.001.

### 3.2 Measurement model fit

Table 3 presents the fit indices for the confirmatory factor analysis (CFA) of the four-factor measurement model. The model demonstrated a good to excellent fit to the data, with all indices meeting or exceeding established thresholds for good model fit (Hu and Bentler, 1999): χ^2^/df = 1.89 (acceptable if < 3.0), RMSEA = 0.05 (good if < 0.06), CFI = 0.96 (excellent if > 0.95), TLI = 0.95 (excellent if > 0.95), and SRMR = 0.05 (good if < 0.08). These results support the discriminant and convergent validity of the measurement model and justify its use in subsequent structural analyses.

**TABLE 3:** Goodness-of-fit indices for the four-factor measurement model.

Model fit index	Observed value	Recommended threshold	Interpretation
χ^2^/df ratio	1.89	<3.0	Acceptable fit
Root mean square error of approximation (RMSEA)	0.05 (90% CI: 0.03–0.06)	<0.08 (good: < 0.05)	Good fit
Comparative fit index (CFI)	0.96	>0.90 (excellent: > 0.95)	Excellent fit
Tucker-Lewis index (TLI)	0.95	>0.90	Excellent fit
Standardized root mean square residual (SRMR)	0.05	<0.08	Good fit

### 3.3 Structural model results

The hypothesized structural model also demonstrated a good fit to the data (Hu and Bentler, 1999): χ^2^/df = 2.15, RMSEA = 0.06 (90% CI: 0.04, 0.07), CFI = 0.94, TLI = 0.93, SRMR = 0.06. All indices met the threshold for acceptable model fit (e.g., CFI/TLI > 0.90, RMSEA/SRMR < 0.08), indicating that the proposed structural relationships are well-supported by the observed data.

**FIGURE 2:**
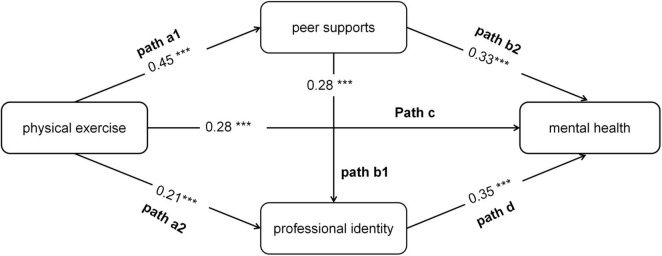
Structural equation modeling results for the dual-chain mediation model (standardized path coefficients shown). The model depicts the direct effects (solid arrows) of physical exercise on peer support, professional identity, and social anxiety, as well as the interrelationships among these psychosocial variables. All path coefficients are standardized estimates (β) ***P < 0.001.

To enhance the clarity of reporting the mediation pathways, we adopted a numerical labeling system for all direct and indirect paths, as suggested in recent methodological guidelines. The specific paths are defined as follows (and are visually represented in Figure 2).

Path a1: Exercise Frequency → Peer Support

Path a2: Exercise Frequency → Professional Identity

Path b1: Peer Support → Professional Identity

Path b2: Peer Support → Mental Health

Path c: Exercise Frequency → Mental Health (Direct Effect)

Path d: Professional Identity → Mental Health

The key indirect effects of interest are:

Indirect Path 1 (Serial): a1 * b1 * d

Indirect Path 2 (Parallel): a2 * d

Indirect Path 3 (Simple): a1 * b2

The structural model results, featuring standardized coefficients (β) for the labeled paths, are presented in Table 4 and Figure 2. The total effect of exercise frequency on mental health was significant (β = 0.28, *P* < 0.001). This total effect was comprised of a significant direct effect (Path c: β = 0.12, *P* < 0.05) and several significant indirect effects, which together accounted for 57% of the total effect.

**TABLE 4:** Direct and indirect effects in the structural equation model (standardized estimates).

Pathway	Standardized coefficient (β)	S.E.	95% CI	*P*-value
**Direct effects**				
Exercise frequency → mental health	0.35	0.04	[0.27, 0.43]	<0.001
Exercise frequency → peer support	0.45	0.03	[0.39, 0.51]	<0.001
Peer support → mental health	0.28	0.03	[0.22, 0.34]	<0.001
Exercise frequency → professional identity	0.21	0.04	[0.13, 0.29]	<0.001
Peer support → professional identity	0.34	0.05	[0.24, 0.44]	<0.001
Professional identity → mental health	0.35	0.04	[0.27, 0.43]	<0.001
**Indirect effects**				
Exercise → peer support → mental health	0.13	0.03	[0.09, 0.19]	<0.001
Exercise → peer support → professional identity → mental health	0.05	0.01	[0.03, 0.08]	<0.001

Crucially, we identified two key mediating pathways as hypothesized:

Indirect Path 1 (the serial mediation pathway: a1 → b1 → d) yielded a significant indirect effect (β = 0.044, 95% CI [0.022, 0.075]), accounting for 15.7% of the total effect.

Indirect Path 2 (the parallel mediation pathway: a2 → d) also showed a significant indirect effect (β = 0.074, 95% CI [0.038, 0.120]), accounting for 26.4% of the total effect.

Additionally, Indirect Path 3 (the simple mediation through peer support alone: a1 → b2) was also significant (β = 0.15, 95% CI [0.080, 0.220]) and represented the largest proportion of the total effect (53.6%).

### 3.4 Moderated mediation model results by academic year

The multi-group structural equation model (MSEM) was employed to test the moderating role of academic year (pre-clinical vs. clinical) on the mediating pathways linking exercise frequency to mental health. The model demonstrated acceptable fit to the data (CFI = 0.95, TLI = 0.94, RMSEA = 0.06, 90% CI [0.05, 0.07], SRMR = 0.07), supporting the validity of group comparisons.

Table 5 presents the standardized path coefficients and group differences. Among direct effects, the path from exercise frequency to mental health (Path c) exhibited a stronger negative association in clinical students (β = 0.32, SE = 0.07, 95% CI [-0.46, -0.18]) compared to pre-clinical students (β = 0.25, SE = 0.06, 95% CI [-0.37, -0.13]; Δβ = 0.07, *P* = 0.04), indicating that physical activity more effectively mitigates anxiety during clinical training. The path from peer support to mental health (Path b2) also showed a significantly stronger protective effect in clinical students (β = 0.36, SE = 0.06) than in pre-clinical students (β = 0.30, SE = 0.05; Δβ = 0.06, *P* = 0.03). Notably, academic year significantly moderates pathways involving professional identity. The path from exercise frequency to professional identity (Path a2) was nearly 1.5 times stronger in clinical students (β = 0.28, SE = 0.06) than in pre-clinical students (β = 0.18, SE = 0.05; Δβ = 0.10, *P* = 0.005). Similarly, the effect of peer support on professional identity (Path b1: Δβ = 0.09, *P* = 0.003) and the protective role of professional identity against mental health (Path d: Δβ = 0.10, *P* = 0.002) were both significantly enhanced in the clinical group. Regarding mediating effects, Indirect Path 3 (the simple mediation pathway: a1 → b2) showed comparable effect sizes between groups (pre-clinical: β = 0.13, 34% of total effect; clinical: β = 0.17, 37% of total effect; *P* = 0.15). In contrast, Indirect Path 1 (the serial mediation pathway: a1 → b1 → d) demonstrated a significant group difference. In clinical students, this pathway explained 18% of the total effect (β = 0.08, SE = 0.02), nearly double the proportion in pre-clinical students (10%, β = 0.04, SE = 0.01; Δβ = 0.04, *P* = 0.008).

**TABLE 5:** Standardized path coefficients and group differences by academic year (pre-clinical vs. clinical).

Pathway	Pre-clinical			Clinical			Group difference	
	β	SE	95% CI	β	SE	95% CI	Δβ	*P*-value
**Direct effects**								
Exercise → mental health	0.30	0.05	[−0.20, −0.40]	0.42	0.06	[0.30, 0.54]	0.12**	0.002
Exercise → peer support	0.42	0.04	[0.34, 0.50]	0.48	0.05	[0.38, 0.58]	0.06	0.12
Peer support → mental health	0.25	0.03	[−0.19, −0.31]	0.32	0.04	[0.24, −0.40]	0.07*	0.04
Exercise → professional identity	0.18	0.05	[0.08, 0.28]	0.28	0.06	[0.16, 0.40]	0.10**	0.005
Peer support → professional identity	0.25	0.04	[0.17, 0.33]	0.34	0.05	[0.24, 0.44]	0.09**	0.003
Professional identity → mental health	0.30	0.05	[0.20, 0.40]	0.40	0.06	[−0.52, −0.28]	0.10**	0.002
**Indirect effects**								
Exercise → peer support → mental health	0.11	0.03	[0.07, 0.19]	0.15	0.04	[0.10, 0.24]	0.04	0.15
Exercise → peer support → professional identity → mental health	0.04	0.01	[0.02, −0.06]	0.09	0.02	[0.05, 0.11]	0.05**	0.008

*P < 0.05, **P < 0.01.

**FIGURE 3:**
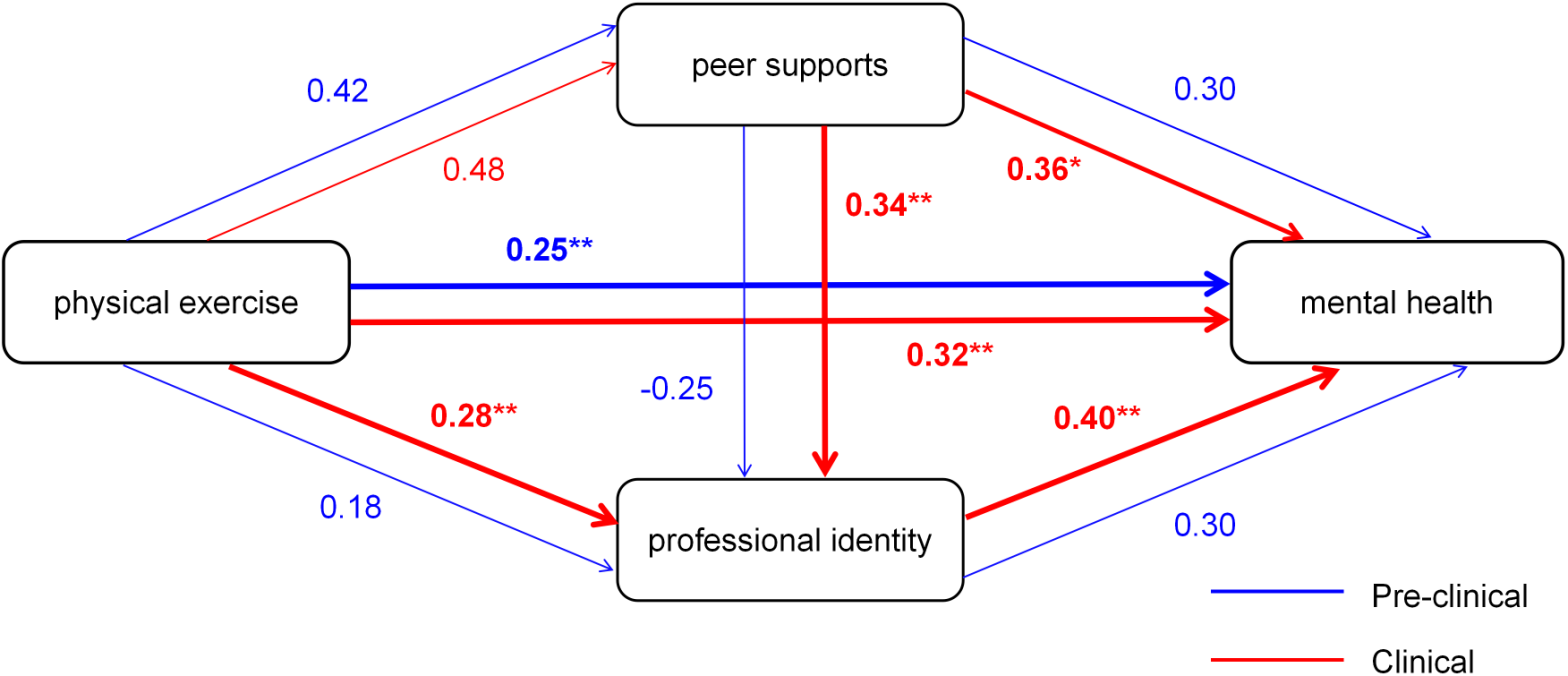
Moderated mediation model of exercise frequency, peer support, professional identity, and mental health, moderated by academic year. The model compares path coefficients between pre-clinical and clinical student groups. Standardized coefficients (β) for each group are displayed on the paths (pre-clinical/Clinical). Significance levels are denoted as: *P < 0.05, **P < 0.01, ***P < 0.001. Paths significantly stronger in clinical students are highlighted in red, and those stronger in pre-clinical students are highlighted in blue.

Figure 3 graphically represents the moderated mediation model. Direct effects are depicted with solid arrows, while mediation pathways are shown as dashed arrows. Pathways with significant group differences (*P* < 0.05) are highlighted in bold, with red arrows indicating stronger effects in clinical students and blue arrows in pre-clinical students. The diagram clearly illustrates that professional identity-related pathways are substantially amplified during the clinical phase, underscoring its emerging importance in linking exercise to anxiety reduction.

## 4 Discussion

This study elucidates the psychosocial mechanisms linking physical exercise to mental health in medical students through a dual-chain mediation model. Our findings demonstrate that the benefits of exercise extend beyond direct physiological effects, operating through two distinct yet interconnected pathways: cultivating peer support and reinforcing professional identity. The following discussion synthesizes these findings theoretically, contextualizes them within existing literature, derives their broader implications, and acknowledges the study’s limitations (Stevens et al., 2021).

### 4.1 Theoretical synthesis: from direct effects to an integrated psychosocial model

Our results provide empirical support for an integrated theoretical framework connecting physical exercise with mental health outcomes. The significant direct effect of exercise frequency on mental health (β = 0.28, *P* < 0.001) aligns with established physiological paradigms, while the identified mediation pathways reveal crucial psychosocial mechanisms.

The serial mediation pathway (exercise → peer support → professional identity → mental health) represents our most theoretically significant finding. This pathway integrates Stress-Buffering Hypothesis with Social Identity Theory, demonstrating that peer support acquired through group-based physical activities serves not merely as a stress buffer but as an active catalyst for professional identity formation. Within exercise-based micro-communities, shared physical challenges and mutual encouragement create environments where medical students socially validate and reinforce their emerging professional roles, transforming group membership into strengthened professional self-concept.

The parallel mediation pathway (exercise → professional identity → mental health) underscores the role of Self-Determination Theory in this process. The development of competence and self-efficacy through mastering physical challenges appears to generalize metaphorically to professional domains, directly enhancing students’ confidence in their clinical roles and capabilities.

The moderating effect of academic year further enriches our theoretical understanding. The significantly stronger mediation role of professional identity among clinical-year students (Δβ = 0.10, *P* = 0.005) suggests that the identity-consolidating function of exercise becomes particularly salient during periods of intense role transition. As students encounter real-world clinical responsibilities, the competencies demonstrated through physical exercise—discipline, perseverance, and resilience—become directly relevant to their professional identity formation, amplifying this pathway’s importance.

### 4.2 Contextual comparison with existing literature

Our findings both converge with and extend the current understanding of exercise benefits in educational contexts. The demonstrated importance of peer support corroborates previous research on social factors in student wellbeing, while providing specificity to how such support is generated and operates. Unlike studies examining social support as a general construct, our model identifies physical exercise as a specific, potent context for developing peer connections that subsequently influence professional development.

The sequential mediation through professional identity represents a novel contribution to the literature on professional identity formation. While previous research has identified various contributors to professional identity, our study uniquely positions physical exercise as an initiator of this process through both social and individual pathways. This expands the conceptualization of professional development beyond formal curricula and clinical experiences to include intentionally designed physical activities.

Compared to studies reporting simple mediation models in exercise psychology, our dual-chain framework offers greater explanatory precision by testing and contrasting multiple mechanisms simultaneously. The finding that both serial and parallel pathways remained significant suggests these are complementary rather than competing explanations, each accounting for unique variance in mental health outcomes.

### 4.3 Theoretical, practical, and methodological implications

Theoretically, our integrated model advances understanding of how exercise benefits operate in professional training environments. By demonstrating that exercise simultaneously activates social support and identity processes, we provide a more comprehensive framework than models emphasizing either mechanism exclusively.

Practically, these findings suggest targeted interventions for medical education. For pre-clinical students, interventions should emphasize peer support activation through structured group activities (e.g., team sports with integrated reflection sessions). For clinical students, programs should leverage the identity pathway by explicitly linking physical challenges to clinical competencies (e.g., “resilience training” that frames workout goals as metaphors for clinical endurance). Additionally, institutional policies could formally recognize such programs as contributing to professional development.

Methodologically, our approach demonstrates the value of testing complex mediation models with multi-group analysis. The use of bootstrap resampling and rigorous fit indices provides a template for future research investigating multiple mechanisms in behavioral interventions.

### 4.4 Limitations and future directions

Several limitations warrant consideration. First, the cross-sectional design precludes causal inference; future longitudinal or experimental studies should establish temporal precedence and causal directionality. Second, convenience sampling may limit generalizability, though our acceptable response rate mitigates this concern somewhat. Third, our measurement of exercise did not capture type or intensity, which may moderate the observed pathways; future research should examine how different exercise modalities preferentially activate specific mechanisms.

The cultural context of our Chinese sample may influence the generalizability of findings. Replication in diverse educational and cultural settings would strengthen the model’s external validity. Finally, incorporating physiological markers (e.g., stress hormones, neuroimaging) could elucidate the bio-behavioral bridges between the psychosocial mechanisms identified here and their physiological substrates.

Despite these limitations, our study provides robust evidence for a dual-pathway model linking physical exercise to mental health in medical trainees. By specifying the mechanisms through which exercise confers psychological benefits, we offer both theoretical advancement and practical guidance for supporting future physicians’ wellbeing

### 4.5 Implications for medical curriculum design

The findings of this study offer clear, actionable guidance for the systematic design of medical curricula. Building upon the validated dual-chain mediation model, we propose several concrete strategies to optimize curricular structures, leveraging physical exercise to activate the key psychosocial mechanisms of peer support and professional identity formation.

#### 4.5.1 Integrating identity-conscious physical activity into the core curriculum

Medical schools should formally incorporate team-based physical activities (e.g., basketball, volleyball, group fitness classes) into mandatory “Physician Wellness and Professional Development” modules, moving beyond optional offerings. Crucially, these activities should be designed with guided reflection sessions. For instance, following an endurance workout, facilitated discussions could focus on how “physical perseverance serves as a metaphor for the resilience required during long clinical shifts or when managing complex patient cases.” This explicitly connects the sense of personal accomplishment gained through exercise to the core competencies of the professional role.

#### 4.5.2 Structuring team-based learning activities

Clinical skills training should be supplemented with team-oriented physical challenges. An example includes designing an “Emergency Response Physical Relay,” where students must collaborate to perform simulated CPR or patient assessment while sequentially completing tasks like shuttle runs or carrying weighted objects. Such activities not only improve physical fitness but also allow students to directly practice and reinforce critical clinical teamwork behaviors—communication, trust, and mutual aid—in a non-clinical setting, thereby solidifying professional identity through action.

#### 4.5.3 Implementing institutionalized peer support systems

Initiatives like “Peer-Supported Wellness Cohorts” or a structured peer mentorship program should be established, pairing students across different academic years to jointly plan and participate in weekly physical exercise. These programs should be strategically activated during high-stress transition periods, such as the start of clinical rotations or examination periods. Within these pairs, senior students not only provide emotional and practical support but also serve as potent professional role models, offering observational learning and a tangible reference for identity formation for their junior counterparts.

#### 4.5.4 Leveraging technology for integrated feedback

The use of wellness tracking applications should be expanded beyond recording steps or workout duration. Apps could be developed or adapted to simultaneously track engagement in group-based activities and interaction frequency within peer networks. Providing integrated feedback, such as, “Your participation in team sports this week not only boosted your physical activity but also strengthened your social connectedness within your academic cohort,” can help students and educators alike monitor developmental trajectories across physical, social, and professional-psychological domains.

## 5 Conclusion

The dual-chain model uncovered in this study provides a clear, actionable blueprint for medical schools seeking to enhance student wellbeing through targeted, theory-informed programming. Moving forward, intervention efforts must extend beyond the generic promotion of physical activity and instead deliberately design initiatives that activate the specific psychosocial mechanisms identified here.

Practically, this entails a shift in curriculum design: (1) Integrating identity-conscious physical activity into the core curriculum, such as by framing team sports as exercises in collaborative care and individual endurance training as a metaphor for clinical perseverance; (2) Structuring peer-supported wellness cohorts, particularly during stressful transition periods like the start of clinical rotations, to leverage the stress-buffering and identity-reinforcing power of shared experience; and (3) Utilizing technology, like wellness apps, to track both physical activity and engagement in these social-professional networks, providing feedback on both fronts.

Ultimately, by reconceptualizing physical exercise not just as a wellness activity but as a foundational component of professional identity formation and peer support, educators can cultivate a generation of physicians who are not only healthier but also more resilient and more deeply connected to their professional roles and to each other.

## Data Availability

The raw data supporting the conclusions of this article will be made available by the authors, without undue reservation.
