# Supplementary material for: Physical exercise-induced mental health benefits in future physicians: a dual-chain mediation of peer support and professional identity formation
Source: Front Psychol. 2025 Dec 3;16:1640506. doi: 10.3389/fpsyg.2025.1640506 (PMC12709623; doi:10.3389/fpsyg.2025.1640506)
Supplement: Supplementary file 1 [file Table_1.docx]

S**upplementary Table S1:** Psychometric Properties of the Measurement Scales

| **Construct** | **Scale Name (Abbreviation)** | **No. of Items** | **Sample Item** | **Response Format** | **Cronbach's α** | **Composite Reliability (CR)** | **Average Variance Extracted (AVE)** | **Factor Loading Range** |
| --- | --- | --- | --- | --- | --- | --- | --- | --- |
| **Physical Exercise** | International Physical Activity Questionnaire-Short Form (IPAQ-SF) | 1 | "During the last 7 days, on how many days did you do moderate-to-vigorous physical activities?" | Days per week | - ^ | - ^ | - ^ | - ^ |
| **Peer Support** | Multidimensional Peer Support Scale (MPSS) | 8 | "I can rely on my peers for encouragement during physical activity." | 1 (Strongly Disagree) to 5 (Strongly Agree) | 0.86 | 0.88 | 0.52 | 0.68 - 0.79 |
| **Professional Identity** | Medical Professional Identity Scale (MPIS) | 10 | "I feel proud to be training as a physician." | 1 (Strongly Disagree) to 7 (Strongly Agree) | 0.91 | 0.93 | 0.58 | 0.72 - 0.85 |
| **Mental Health - Burnout** (Composite) | Maslach Burnout Inventory-Student Version (MBI-SV) | 15 | "I feel emotionally drained by my studies." (Emotional Exhaustion) | 0 (Never) to 6 (Everyday) | 0.89 | 0.91 | 0.55 | 0.65 - 0.82 |
| **Mental Health - Psychological Well-Being** | Ryff's Scales of Psychological Well-Being (18-item version) | 18 | "I am confident about my own opinions, even if they are contrary to the general consensus." (Autonomy) | 1 (Strongly Disagree) to 6 (Strongly Agree) | 0.92 | 0.94 | 0.57 | 0.70 - 0.84 |

^ Physical exercise was assessed using a single-item metric (frequency). Conventional reliability (α) and construct reliability (CR, AVE) metrics are not applicable to single-item measures.

**Supplementary Table S1.** Psychometric Properties of the Measurement Scales
